# Influenza Viral Manipulation of Sphingolipid Metabolism and Signaling to Modulate Host Defense System

**DOI:** 10.1155/2014/793815

**Published:** 2014-01-23

**Authors:** Madhuvanthi Vijayan, Bumsuk Hahm

**Affiliations:** ^1^Departments of Surgery and Molecular Microbiology & Immunology, University of Missouri-Columbia, Columbia, MO 65212, USA; ^2^University of Missouri-Columbia, One Hospital Drive, Medical Sciences Building, NW301C, Columbia, MO 65212, USA

## Abstract

Viruses attempt to create a distinctive cellular environment to favor viral replication and spread. Recent studies uncovered new functions of the sphingolipid signaling/metabolism during pathogenic virus infections. While sphingolipids such as sphingomyelin and ceramide were reported to influence the entry step of several viruses, sphingolipid-metabolizing enzymes could directly alter viral replication processes. Influenza virus was shown to increase the level of sphingosine kinase (SK) 1 to promote virus propagation. The mechanism involves regulation of intracellular signaling pathways, leading to the amplification of influenza viral RNA synthesis and nuclear export of viral ribonucleoprotein (RNP) complex. However, bovine viral diarrhea virus inhibits SK1 to enhance the efficacy of virus replication, demonstrating the presence of virus-specific strategies for modulation of the sphingolipid system. Therefore, investigating the sphingolipid metabolism and signaling in the context of virus replication could help us design innovative therapeutic approaches to improve human health.

## 1. Introduction

**Figure 1:**
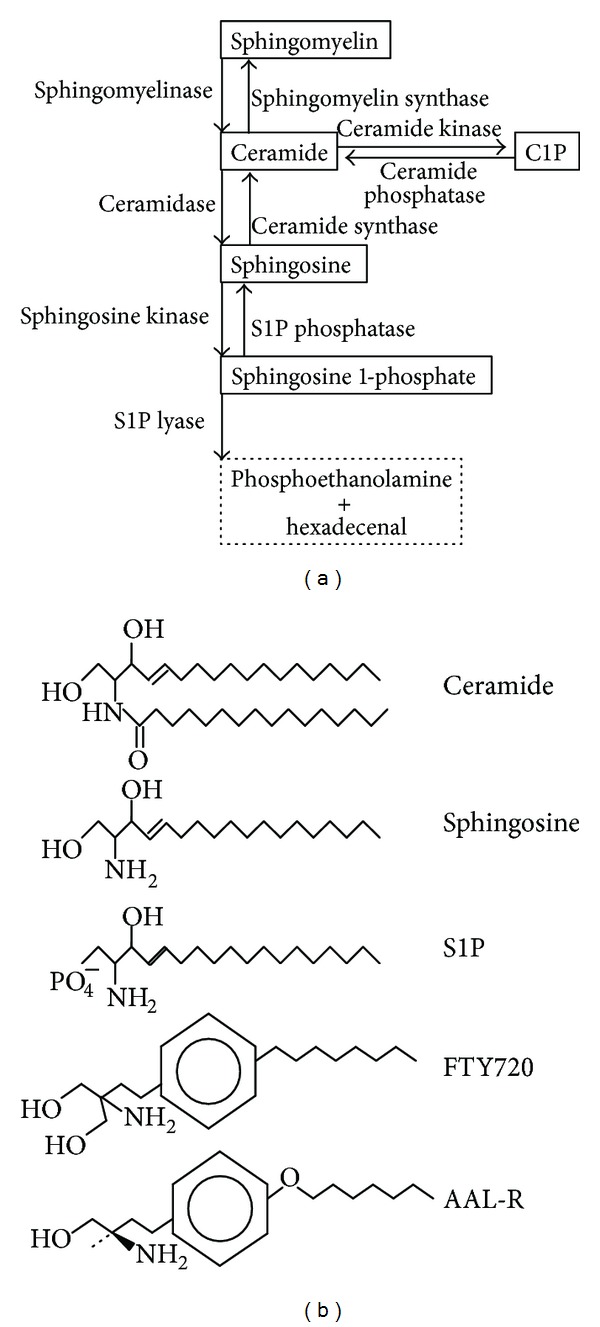
The sphingolipid system. (a) The pathway of sphingolipid synthesis and degradation by catalytic enzymes. (b) Shown are chemical structures of the sphingolipids (ceramide, sphingosine, and S1P) and the sphingosine analogs (FTY720 and AAL-R).

Over the last twenty years, the field of sphingolipid system has received enormous attention of scientists and clinicians, since the sphingolipids have numerous important biological functions and are therapeutically applicable to the treatment of human diseases [1–5]. The sphingolipids are bioactive mediators and include sphingomyelin, ceramide, ceramide-1-phosphate, sphingosine, and sphingosine 1-phosphate (S1P) (Figure 1(a)). The levels of these sphingolipids are tightly regulated by cellular metabolizing enzymes to maintain normal cellular physiological conditions [6, 7]. The sphingosine analog FTY720 (fingolimod, gilenya) (Figure 1(b)) transiently represses the exit of T lymphocytes from lymph nodes and acts as an agonist of S1P receptors (S1P1 and S1P3–5) except for S1P receptor 2 [8, 9]. FTY720, which is a derivative of myriocin that is found in a fungus and used in Chinese medicine, is the first oral therapeutic agent approved by the US FDA to treat multiple sclerosis (MS) [10, 11]. Also, the FTY720 derivative AAL-R or S1P receptor 1 agonist was shown to alleviate influenza virus-induced immunopathology [12–15] and the detailed information about this is found in excellent review articles written by Oldstone and Rosen's groups [16–18].

Pharmacologic inhibitors of sphingosine kinase (SK) have therapeutic potential to suppress cancer progression [4, 19]. Further, SK and S1P lyase were shown to regulate virus replication [20, 21]. Importantly, several virus infections alter SK expression or activation in the cells, suggesting that these viruses regulate the sphingolipid system to promote their own replication and propagation [22–26]. This review is focused on our recent findings on influenza viral regulation of S1P-metabolizing enzymes. This report will also document the interaction of other viruses with the sphingolipid system in comparison.

## 2. Regulation of Influenza Virus Replication by S1P-Metabolizing Enzymes

### 2.1. Influenza Virus

Influenza virus continues to pose a worldwide threat to humans by causing approximately 500,000 deaths annually [27]. Influenza virus belongs to the *Orthomyxoviridae* family and is divided into three types, A, B, and C [28, 29]. Influenza A viruses (H1N1 and H3N2) and influenza B virus are currently circulating in humans to cause annual epidemics [30]. Importantly, several influenza A viruses that infect birds or pigs were shown to undergo antigenic shift and genetic mutation and thus acquire the capacity to infect humans [31]. Some of them have caused influenza pandemics such as that 1918 Spanish flu, Asian flu, Hong Kong flu, and 2009 H1N1 flu. Currently, avian influenza viruses such as H5N1 and H7N9 flu sporadically infect humans with high mortality rates [32, 33]. These dangerous viruses have not caused influenza pandemic, since they have not acquired the ability to transmit among humans yet.

Influenza virus interacts with sialic acids *α*2–6-linked to galactose to infect human cells [31]. Viral M2 protein acts as an ion channel to cause viral fusion with the endosome and uncoating for the release of the viral ribonucleoprotein (RNP) complex into cytoplasm [34]. Subsequently, the RNP complex is imported into the nucleus and viral RNA transcription and replication initiate to produce viral proteins and amplify viral RNAs. Influenza virus steals the 5′ cap from cellular pre-mRNAs, referred to as “cap snatching” [35] and the viral polymerase complex composed of PB1/PB2/PA generates mRNA for translation of viral proteins. Since viral RNAs are synthesized in the nucleus, the viral RNP complex that consists of mainly viral RNAs and nucleoprotein (NP) needs to be exported to the cytoplasm for the assembly of new progeny viruses [36]. The nuclear export of viral RNPs involves the function of viral NS2 and M1 and the process is mediated by cellular export machinery of chromosome region maintenance 1 (CRM1, exportin) [37]. Viral assembly occurs at the plasma membrane and thus no progeny viruses are found inside cells [36]. The NA activity to cleave sialic acid residues is essential for the prevention of virus aggregation and the release of infectious progeny virions.

Current antiviral drugs were designed based on their inhibitory activity of virus replication by blocking the function of viral proteins such as NA and M2. However, influenza virus can change their genetic sequences to escape the action of antivirals [38, 39]. Indeed, many seasonal influenza virus strains as well as pathogenic H5N1 avian flu strains were shown to be resistant to current treatments [38, 39]. Therefore, other intervention approaches are needed to evade the occurrence of viral genetic changes.

### 2.2. The Role of S1P Lyase during Influenza Virus Replication

**Figure 2:**
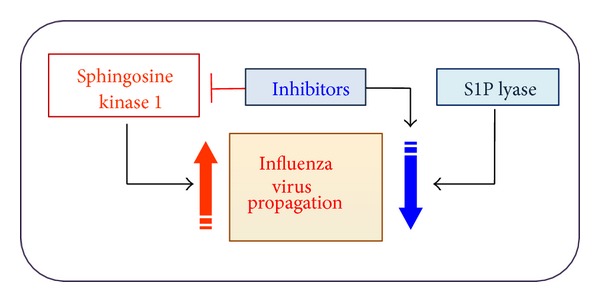
Modulation of influenza virus amplification by sphingosine kinase 1 and S1P lyase. SK1 increases influenza virus replication, whereas S1P lyase inhibits virus propagation. Manipulation of SK1 such as SK1 inhibition suppresses production of infectious influenza viruses.

S1P lyase degrades S1P in an irreversible manner and is therefore important for regulation of sphingolipid levels and their functions [6]. Interestingly, overexpression of S1P lyase inhibited the expression of influenza virus proteins and the production of infectious progeny viruses (Figure 2). The suppressive effect was reversed when S1P lyase expression was downregulated by using siRNA. Similar increase of virus replication was observed when the cells were treated with the inhibitor of S1P lyase (unpublished result). Therefore, S1P lyase expression or activation negatively regulates the process of influenza virus replication. So far, to our knowledge, there is no report published about the regulation of virus replication mediated by S1P lyase. Therefore, it is unknown if this is a specific controlling mechanism for influenza virus or if S1P lyase regulates the replication process of other viruses.

The molecular mechanism for S1P lyase inhibition of influenza replication seems to involve the activation of ERK signaling and JAK/STAT signaling pathways, since S1P lyase-overexpression induced earlier activation of ERK, along with earlier and stronger activation of STAT1/STAT2 upon influenza virus infection [21]. Previously, influenza virus was known to activate ERK in a biphasic manner, which is critical for efficient virus replication [40]. It is possible that S1P lyase dysregulates influenza virus-induced activation of ERK MAPK. Rapid and augmented activation of STAT1/STAT2 indicates increased antiviral type I interferon (IFN) signaling [41]. However, S1P lyase overexpression did not increase the level of type I interferon (IFN) expression upon infection. Therefore, it remains to be further studied how S1P lyase exerts its antiviral activity against influenza and how the type I IFN signaling pathway of JAK/STAT is linked to S1P lyase function upon infection. Generation of other experimental systems to carefully control S1P lyase expression/activation will facilitate the study on the molecular mechanisms by which S1P lyase mediates the restriction of influenza virus replication.

### 2.3. Proinfluenza Viral Function of Sphingosine Kinase 1

Whereas S1P lyase induces degradation of S1P, sphingosine kinase (SK) generates S1P from sphingosine (Figure 1). Overexpression of SK1 was shown to increase cellular permissiveness to influenza virus infection by allowing enhanced viral protein synthesis and amplification of progeny viruses (Figure 2). In contrast, when lung epithelial cells were treated with pharmacologic inhibitors of SK or when the cells were modified to express reduced level of SK1 by using siRNA, influenza virus replication was suppressed [24]. These results led us to claim that SK1 is required for efficient replication of influenza virus. The inhibition of influenza virus replication by the inhibitor of SK was observed in several cell types such as A549 (human lung epithelial cells), NCI-H358 (human lung epithelial cells), HEK293 (human embryonic cells), and MDCK cells (canine kidney epithelial cells). Further, the inhibition was observed when the cells were infected with influenza A/WSN/33 virus (H1N1) or influenza A/Hong Kong/8/68 (H3N2) virus. Yet, it remains to be studied if similar inhibition occurs when cells are infected with highly pathogenic avian influenza viruses and pandemic viruses.

#### 2.3.1. Regulation of SK1 Level/Activation by Influenza Virus Infection

**Figure 3:**
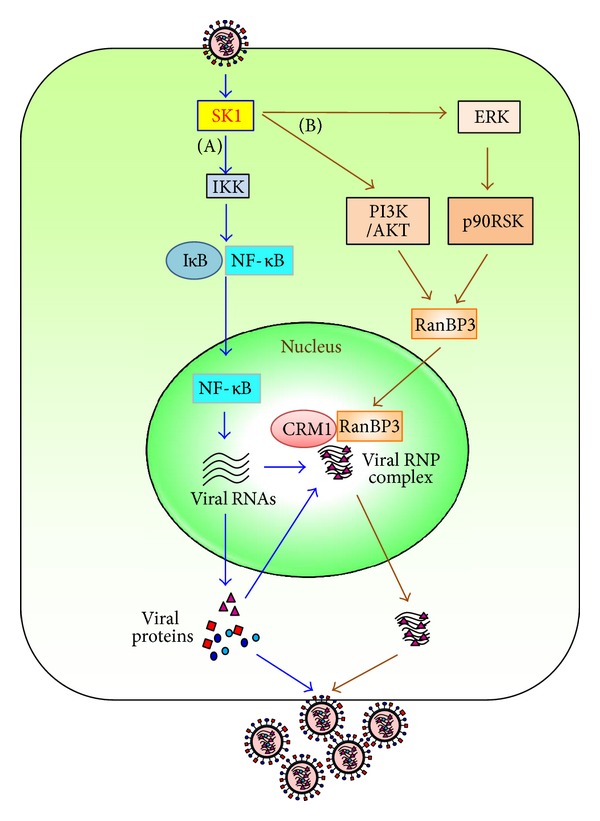
Model for the proviral function of SK1 in influenza virus replication. The figure is adapted from Seo et al. [24].

Influenza virus infection increased the level of SK1 in the cells (Figure 3). Further, phosphorylated SK1, which is the activated form of SK1, was shown to increase by the infection [24]. Because SK1 increases the efficiency of influenza virus replication, virus-induced activation/upregulation of SK1 should create a favorable environment for influenza virus replication.

The mechanism by which influenza virus induces SK1 activation/upregulation is unknown. It was reported that toll-like receptor (TLR)4 engagement induced activation and transcriptional upregulation of SK1 in macrophages [42]. This suggests that recognition of pathogenic molecules by innate immune system may regulate SK activation/expression. However, when human macrophages were stimulated with R848 (ligand for TLR7/8), activation of SK1 was not observed in their experimental system [42]. It is also possible that virus-induced cytokines such as TNF increase the activation of SK1. Indeed, TNF binding to its cognate receptor induced SK1 activation followed by activation of NF-*κ*B signaling [43, 44]. Involvement of viral proteins in this process is unknown. Therefore, further studies are required to investigate the mechanism by which influenza virus activates and increases the level of SK1.

#### 2.3.2. Influenza Viral Life Cycle Regulated by SK Inhibition

We employed the inhibitor approach to delineate the cellular and molecular mechanisms by which SK inhibition modulates influenza virus replication. SK inhibition did not alter the entry of influenza virus into the cells but restricted the amplification of viral RNAs and proteins, resulting in the marked decrease of influenza virus propagation (Figure 3). The synthesis of viral RNAs was minimally affected at one or two hours after virus infection, but SK inhibitor restrained the amplification of both positive and negative RNAs at five hours following infection [24]. Furthermore, SK inhibition impaired the nuclear export of ribonucleoprotein (RNP) complex (Figure 3). This was independent of the inhibition of viral protein expression, since the inhibition of RNP export occurred even when virus-infected cells were treated with a protein synthesis inhibitor. Therefore, two distinctly different inhibitory stages were identified: RNA/protein synthesis and export of the viral RNP complex. Interestingly, sphingomyelin synthase-1 was shown to be critical for the transport of influenza viral HA and NA to the plasma membrane [45]. Therefore, it is also possible that SK1 modulates another step of influenza virus replication.

SK inhibition interfered with the export of viral NP from the nucleus to the cytoplasm. It also inhibited the export of viral NS2 and M1, which are known to be important for the export process of viral RNP complex in the infected cells [46]. However, the localization of other viral proteins such as NS1 and M2 that are unrelated to the export process was not altered by SK inhibition [24]. Influenza virus was known to utilize CRM1-mediated cellular export signaling for the export of viral RNP complex [37]. The CRM1-mediated export of many cellular proteins necessitates the activation of Ran binding protein 3 (RanBP3), although RanBP3-independent CRM1-mediated pathway exists [47, 48]. Therefore, the role of RanBP3 in the influenza virus replication was investigated by us as well as by other investigators [48]. The inhibition of RanBP3 expression minimally affected protein expression of influenza virus [24]. However, the expression of RanBP3 was critical for nuclear export of influenza viral RNP complex and production of infectious influenza viruses. Importantly, influenza virus infection increased the phosphorylation of RanBP3 and SK inhibition suppressed virus-induced activation of RanBP3 [24]. Therefore, RanBP3 could be another cellular target protein for blocking virus replication and possibly treating influenza.

#### 2.3.3. Cellular Signaling Pathways Regulated by SK Inhibition during Influenza Virus Replication

Influenza virus was reported to activate multiple signal transduction pathways to make the intracellular environment extremely affordable for viral propagation [49]. SK inhibition was shown to interfere with such proviral pathways to inhibit influenza virus replication. This includes the cellular NF-*κ*B pathway, which is essential for influenza viral RNA synthesis and the CRM1/RanBP3 nuclear export pathway, which facilitates the transport of viral RNP complexes from the nucleus to the cytoplasm of the infected cell [24]. Thus, SK inhibition seems to interfere with two very important stages in the intracellular life cycle of influenza virus.

*(a) The NF-*κ*B Pathway.* The NF-*κ*B family of transcription factors is a collection of proteins that are evolutionarily conserved and linked to a multitude of biological processes ranging from cell growth, cell differentiation, cellular stress response, induction of inflammatory responses and regulation of immune pathways [50, 51]. Because of the central role of the NF-*κ*B pathway in orchestrating diverse cellular and physiological responses, dysregulation of this pathway can lead to abnormalities such as cancer, autoimmunity, and several other pathological outcomes [52]. For the same reason, viruses exploit this pathway to reprogram the cellular milieu for their own replication advantage [53]. The following sections summarize the interplay between viruses and the NF-*κ*B signaling pathway in addition to discussing some general aspects of the NF-*κ*B pathway.

**Figure 4:**
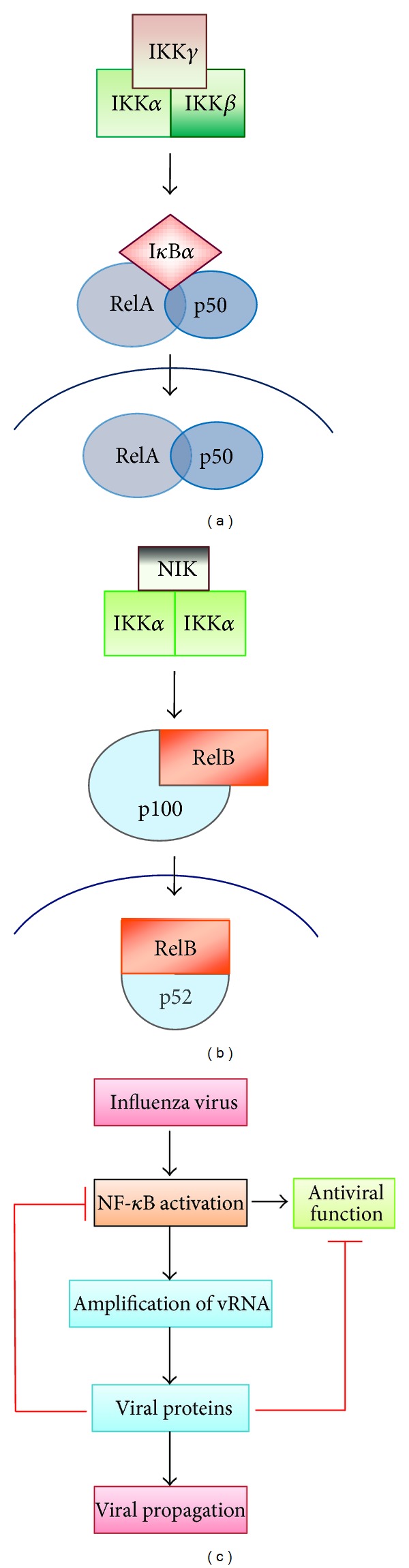
NF-*κ*B signaling pathway and its regulation by influenza virus. (a) The canonical pathway involves activation of the IKK complex, IKK-mediated I*κ*B*α* phosphorylation, and subsequent degradation, resulting in nuclear translocation of the heterodimer RelA(p65)/p50. (b) The noncanonical pathway is dependent on phosphorylation-induced p100 processing. This leads to activation/translocation of the RelB/p52 complex. (c) Influenza virus activates the NF-*κ*B pathway to facilitate the amplification of vRNAs and production of progeny viruses. Newly synthesized influenza viral proteins antagonize the activation of NF-*κ*B signaling and inhibit the function of antiviral proteins or immunoregulatory molecules induced by the pathway.

*Canonical and Noncanonical NF-*κ*B Pathways*. There are 2 types of NF-*κ*B pathways that are known to exist, that is, the classical or the canonical pathway and the alternative or the noncanonical pathway (Figures 4(a) and 4(b)). These two pathways differ in their mode of activation and the signaling intermediates and have distinct functional outcomes [54, 55]. The classical pathway is considered as the major pathway that is commonly triggered by many biological stimuli. Some well-known examples are the ligand-activated TNFR1/2 pathway, TLR4 pathway, and the T cell receptor pathway [56]. On the other hand, the noncanonical pathway is activated in response to lymphotoxin *β* receptor, B-cell-activating factor belonging to the TNF family receptor, CD40, and CD30 [57]. The canonical pathway depends on the activation-induced degradation of I*κ*B*α* (Figure 4(a)). I*κ*B*α* is phosphorylated in an I*κ*B kinase *β* (IKK*β*) and IKK*γ*-dependent manner, resulting in the phosphorylation and subsequent nuclear translocation of p65 (RelA)-containing heterodimers such as p50/p65 [58]. The dimers are transcriptionally active and can bind to promoter and enhancer regions of target genes. In contrast, the noncanonical pathway depends on the NF-*κ*B-inducing kinase (NIK)-mediated activation of IKK*α*. Activated IKK*α* homodimerizes and phosphorylates p100. This leads to ubiquitination and proteasomal processing of p100 to generate the transcriptionally active p52 containing dimers such as p52/RelB, as illustrated in Figure 4(b).

*Interplay between Viruses and the NF-*κ*B Pathway*. It is well established that NF-*κ*B signaling is an important innate antiviral immune response to virus infections [59]. Hence, it is not surprising that NF-*κ*B pathway remains as an obvious target to viral pathogens. In this section, we will summarize what is previously known about influenza virus-mediated activation of NF-*κ*B pathway and further document our findings on how SK1 fits into this knowledge. We will also discuss the impact that the NF-*κ*B pathway has on the life cycle of several other viruses.

Many viruses are known to encode for proteins to block/antagonize the NF-*κ*B pathway to subvert the host antiviral response. Some examples include the influenza NS1 protein [60] and hepatitis C virus (HCV) core proteins [61]. Recently, influenza NS1 was also shown to physically bind to IKK*α* and IKK*β* to interfere with IKK function to prevent the induction of antiviral genes [62]. Interestingly, NS1 was shown to interfere with both the classical and the alternative activation of the NF-*κ*B pathway [62]. Similar to influenza NS1, the core protein of HCV was shown to bind and inhibit the IKK*β* kinase activity, to prevent NF-*κ*B activation [61]. Along the same lines, measles virus, a member of *Paramyxoviridae* family, was also reported to inhibit NF-*κ*B signaling using its V protein, which binds to the p65 subunit, thereby preventing the nuclear translocation of p65 and the activation of target genes [63].

Contrary to the above examples, many viruses are also known to incorporate the NF-*κ*B pathway into their life cycle to enhance their replication/pathogenesis. A well-known example to this is human immunodeficiency virus (HIV) infection. The enhancer region of the HIV-1 long terminal repeat contains NF-*κ*B binding sites that mediate HIV gene expression [64]. Certain tumor viruses are known to activate NF-*κ*B in a constitutive manner to facilitate oncogenic transformation of infected cells. For example, Epstein-Barr virus (EBV) induces in vitro transformation of B cells by activating the NF-*κ*B pathway, which is mediated by its protein, LMP-1 [65].

The proviral facet of NF-*κ*B pathway during influenza virus replication has been well documented by many groups that have shown that an active NF-*κ*B pathway is a pre-requisite for the establishment of a productive infection of host cells by influenza virus [66–69]. More recently, the exact role of the NF-*κ*B pathway in aiding influenza virus replication was attributed to its role in promoting influenza viral RNA synthesis [66]. It was shown that NF-*κ*B signaling was necessary for the synthesis of vRNA (viral RNA) but not the cRNA (complementary RNA) or mRNA (messenger RNA) of influenza virus. Thus, the NF-*κ*B pathway was shown to differentially regulate influenza vRNA synthesis by preferentially affecting replication process from cRNA [66]. In our study, we found that SK inhibition reduced virus-induced phosphorylation of IKK*α*/*β*, an upstream signaling molecule of NF-*κ*B [24]. Consistent with this, a similar reduction in the phosphorylation and nuclear translocation of the active p65 subunit of NF-*κ*B was observed. Furthermore, SK inhibition reduced transcription from NF-*κ*B promoter, demonstrating that SK inhibitor treatment reduces virus-induced NF-*κ*B activation, which subsequently leads to the inhibition of viral RNA synthesis. These findings illustrate how influenza virus utilizes the enzyme SK1 as an intermediate to activate the NF-*κ*B pathway (Figure 3). The SK1-NF-*κ*B pathway triggered upon influenza virus infection of host cells aids in the enhancement of viral replication and propagation.

It is important to note that the NF-*κ*B pathway could play dual divergent roles in the replication cycle of viruses as exemplified by influenza virus (Figure 4(c)). Thus, it appears that some viruses induce a certain level of NF-*κ*B activation, which is possibly under the temporal/spatial regulation, while inhibiting its typical antiviral activity that would eventually interfere with efficient virus replication. From the examples described above, it becomes clear that the manner in which the NF-*κ*B pathway is being modulated by viral pathogens could directly depend on the nature of the viruses including the mechanism of replication, mode of infection (acute or persistent), and viral pathogenesis.

*(b) Cellular Signaling Pathways to Increase Nuclear Export of Viral RNP Complex*. Cellular signaling proteins such as ERK/RSK and AKT can mediate activation of RanBP3 [70]. This was also proved to be the case upon influenza virus infection, since the inhibition of AKT or ERK MAPK signaling suppressed the phosphorylation of RanBP3 following influenza virus infection [24]. Importantly, SK inhibition repressed activation of ERK MAPK and AKT upon influenza virus infection, resulting in the inhibition of virus-induced activation of RanBP3 and CRM1/RanBP3-mediated conveyance of viral RNP complex to the cytoplasm (Figure 3) [24].

The relationship between SK and ERK MAPK has been studied in multiple experimental conditions. SK1 activation was shown to be crucial for TNF or VEGF-induced ERK activation [71]. Further, ERK is also important for the activation of SK1 [72], suggesting that these two kinases regulate each other's activation in a positive manner.

It is unclear if the type I IFN signaling pathway is involved in the mechanism for SK inhibition-mediated suppression of influenza virus propagation. When the SK inhibitor was supplied to the culture after infection, the increased activation of STAT1/2 was not observed (unpublished result). This suggests that SK inhibition does not activate the JAK/STAT type I IFN signaling pathway. This is different from the mechanism for SPL overexpression-mediated inhibition of virus replication, as SPL increased STAT1/2 activation following influenza virus infection [21].

## 3. Interaction of Sphingosine Kinase with Other Viruses

**Figure 5:**
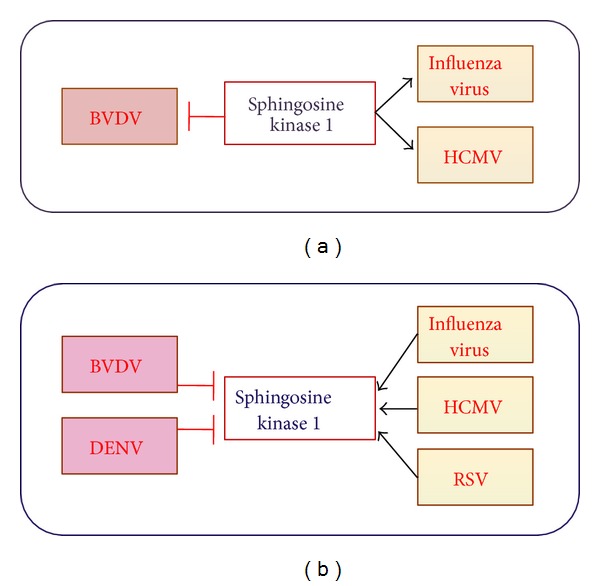
Interaction between SK1 and viruses. (a) SK1 inhibits BVDV replication, whereas SK1 heightens cellular susceptibility to the infection with influenza virus or HCMV. (b) BVDV and DENV decrease SK1 expression/activation, whereas influenza virus, HCMV, and RSV increase SK1 level/activation.

While the role of SPL has not been studied in other virus infections, SK1 has been proposed to modulate the replication of other viruses (Figure 5(a)). Furthermore, several viruses were shown to regulate the level or activity of SK1 enzyme (Figure 5(b)).

Similar to influenza virus, human cytomegalovirus (HCMV) increases SK1 activity and SK1 expression [22]. SK1 seems to contribute to the efficient virus replication, as blockade of SK1 expression decreased immediate early protein IE1 and overexpression of SK1 elevated the expression of IE1. However, the expression of the HCMV early protein UL44 was not affected by the modulation of SK1 expression, suggesting that a specific regulatory mechanism exists. In contrast to influenza virus or HCMV, bovine viral diarrhea virus (BVDV), which is classified in the *Flaviviridae* family, decreased SK1 activity, and the inhibition of SK1 by using the SK-specific inhibitor or siRNA targeting of SK1 resulted in the promotion of BVDV replication [26]. Interestingly, BVDV NS3 protein was shown to directly interact with SK1 and suppress the enzymatic activity of SK1, which was independent of the catalytic activity of NS3 protease. However, NS3 of HCV, which also belongs to the *Flaviviridae* family, did not inhibit SK1 activity [26].

Respiratory syncytial virus (RSV) increased the activity of SK1 and the mRNA expression of SK1 as well. Further, the heightened SK1 activation appeared to mediate RSV-induced activation of ERK MAPK and Akt and thus regulate the cell survival pathway upon infection [23]. However, the detailed mechanism and the role of SK1 activation in RSV replication or pathogenesis require further investigation. Regulation of SK1 activity or expression does not seem to affect the replication of dengue virus type-2 (DENV-2). However, the 3′ untranslated region of DENV-2 was shown to decrease the kinase activity of SK1 [25]. The continued study on DENV-2-mediated regulation of SK1 and its function in DENV-induced inflammatory responses could increase our understanding of DENV pathogenesis.

## 4. Perspectives

**Figure 6:**
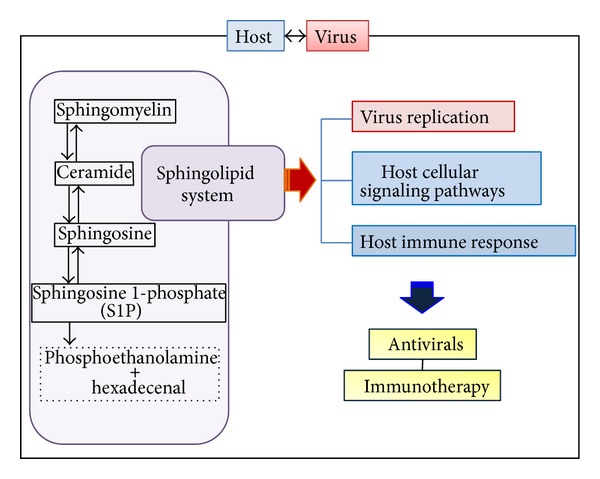
Diagram depicts the sphingolipid system regulating virus replication and host defense and signaling. The study of the sphingolipid metabolism/signaling during viral infection could allow us to develop new therapeutic approaches to clear virus infections.

The sphingolipid system was shown to modulate virus replication and affect host cellular signaling pathways that are important for virus propagation (Figure 6). Additionally, the sphingolipid signaling could regulate host innate and adaptive immune responses to the infection [73–76]. Furthermore, pathogenic viruses were reported to change the level of sphingolipids or activation of sphingolipid-metabolizing enzymes for their benefit. Therefore, understanding the interplay between the virus and sphingolipid metabolism and signaling would help us design novel strategies to overcome viral mechanisms to survive in the cells and ultimately to cure viral diseases. Obviously, extensive studies on sphingolipid-virus interaction and the chemical medicinal research to develop new small molecules that regulate sphingolipid metabolism would be needed to this end.
